# Effect of Electroacupuncture at Different Acupoints on the Expression of NMDA Receptors in ACC and Colon in IBS Rats

**DOI:** 10.1155/2019/4213928

**Published:** 2019-02-03

**Authors:** Li-hua Tan, Kai-ge Li, Yan-ying Wu, Meng-wei Guo, Yin Lan, Shan Wang, Wen-lian Zhu, Xiao-xuan Ren

**Affiliations:** ^1^College of Acupuncture and Massage, Beijing University of Chinese Medicine, Beijing, China; ^2^Maternal and Child Health-Care Hospital of Yichang, Hubei, China; ^3^Beijing Kang Yide Combination of Chinese Traditional and Western Medicine Lung Hospital, China; ^4^Traditional Chinese Medicine of Huairou Hospital in Beijing, China

## Abstract

**Objective:**

To observe the effects of electroacupuncture (EA) at different acupoints on the expression of N-methyl-D-aspartate receptor (NMDA receptor ) and behaviors in irritable bowel syndrome (IBS) rats.

**Methods:**

Wistar rats were randomly divided into blank control group (blank group, n=10) and model preparation group (n=50); experimental rat model of IBS was established by the “neonatal maternal separation and acetic acid enema” combined with “colorectal distension stimulation” method. A total of 50 IBS rats were randomly assigned to five groups of 10 each: model group, Yintang (GV29) group, Neiguan (PC6) group, Tianshu (ST25) group, and Zusanli (ST36) group. Rats in four treatment groups, aged 9 weeks old, were treated with EA by HANS with a sparse-dense wave with a frequency of 2/100 Hz, current of 0.1-0.3mA, and 20 min/stimulation, every other day for a total of 5 sessions. After treatment, the abdominal visceral sensitivity was evaluated by abdominal withdrawal reflex (AWR), and the psychological and emotional behavior of rats were evaluated by the open-field test (OFT). The expression of NMDA receptors in anterior cingulate cortex (ACC) was detected by Quantitative Real-time PCR, and the positive expression of NMDA receptors in colon was detected by immunohistochemistry.

**Results:**

The IBS rat's abdomen is more sensitive and irritable; NR1, NR2A, and NR2B in ACC and NR1 and NR2B in colon of rats significantly increased in the model group versus the normal group (P<0.01) and were inhibited in all treatment groups (P<0.01, P<0.05). Additionally, NR2A and NR2B in ACC reduced more in GV29 group (P<0.01) than in other treatment groups (P all<0.05) compared with the model group. The expression of NR2B in colon was significantly inhibited in ST36 group (P<0.01) and inhibited in GV29 group and ST25 group (P all <0.05) compared with the model group. And the expression of NR2B in colon was more inhibited in ST36 group than in PC6 group (P<0.01).

**Conclusions:**

EA at different acupoints could obviously relieve abdominal pain and abnormal behaviors in IBS rats in different degrees of effects. The effect of abdominal pain-relief, from greatest to least, is ST25, ST36, GV29, and PC6, while the effect of relieving abnormal behaviors caused by IBS, from greatest to least, is GV29, PC6, ST36, and ST25. There are significant differences in the expressions of NMDA receptors in ACC and colon among different acupoints. This difference should be related to the location distribution and indications of acupoints.

## 1. Introduction

Acupuncture and moxibustion believe that stimulating acupoints can specifically regulate the corresponding visceral organs, which is different from nonacupoints and other acupoints. This difference is the acupoint specificity [1]. The specificity of acupoint effect is one of the important bases for acupoint selection along meridians. Moreover, a large number of clinical practices [2–4] and experimental studies [5, 6] have confirmed that acupoint effect has specificity. Previous studies on the effect specificity of acupoints have been conducted to observe the therapeutic effects of different acupoints on the same symptoms of a disease [3, 7]; however, the curative effect of different points on different symptoms of a disease is rarely reported. In order to further verify the specificity of meridian effect, this experiment selected different parts of acupoints with different functions by syndrome differentiation and acupoint selection along meridians to observe whether they have different regulating effects on abdominal pain and abnormal mental disorder in irritable bowel syndrome (IBS).

Irritable bowel syndrome (IBS) is a typical psychosomatic disease manifested as abdominal pain or discomfort, stool irregularities, and bloating, as well as other somatic, visceral, and psychiatric comorbidities [8]. As a multifactorial disease, the underlying pathogenesis of IBS is complex including mental disorders, brain-gut axis dysfunction, and visceral paresthesia. Different etiologies and pathogenesis can influence each other, and the brain-gut axis [9] plays a key role in the interaction of various factors.

N-methyl-D-aspartate receptor (NMDA receptor) is an important excitatory ionotropic glutamate neurotransmitter in the central nervous systemand is associated with mood disorders and psychosis-schizophrenia [10, 11]. NMDA receptor plays a critical role in the neuroplasticity of nociceptive networks, and there is evidence [12–14] proving its involvement in chronic stress-induced visceral hyperalgesia. It has also been reported that NMDA receptor was associated with the formation of high intestinal sensitivity of IBS [15]. Whether there is a close relation between intestinal sensitivity and mental disorders in the pathogenesis of IBS, or whether they play a key role in acupuncture regulation, is to be studied.

Based on the holistic review and syndrome differentiation in Traditional Chinese Medicine (TCM) acupoints with different functions should be selected according to the different diseases and syndromes, and then the curative effect is remarkable [16, 17]. In classical acupuncture, acupoints with effects of tranquilizing the mind, relieving pain, and regulating the functions of stomach and intestines all showed satisfied effect on IBS [18]. However, the specific effect of different acupoint on IBS and its underlying mechanism is still unclear. Therefore, this study selected four commonly used acupoints for IBS including Yintang (GV29), a sedative point, Tianshu (ST25), a point for regulating intestines and bowels, and Neiguan (PC6) and Zusanli (ST36), two points with satisfied effect for both physical and mental diseases, to compare the effects on the behavior and the expression of NMDA receptors in ACC and colon of IBS rats with abdominal pain and emotional disorder. The purpose of this study is to explore the effect and difference of acupoints distributed in head, trunk, and limbs with different therapeutic effects on different symptoms of physical and mental diseases.

## 2. Methods

### 2.1. Animals and Grouping

Eight cleaning Wistar pregnant rats were provided by Beijing Weitong Lihua Experimental Animal Technology Co. Ltd., certificate number: SCXK- (in 2012-004). The rats were fed in the room with temperature of 23±2°C and humidity of about 50%, 12h alternating between day and night, eating and drinking in free. The neonatal rats were randomly divided into blank control group (blank group (n=10)) and model preparation group (n=50).

### 2.2. Irritable Bowel Syndrome Model

The IBS model (n=50) was established by the “neonatal maternal separation and acetic acid enema” combined with the “colorectal distension stimulation” method. The neonatal rats were isolated with mother rats 3h/d from 2 to 21 days after birth. Stimulation was given to the colon by using 0.5% acetic acid from 8^th^ day to 21^th^ day; the central venous catheter (1 mm in diameter) lubricated with paraffin oil was inserted into the colon through the anus for 2 cm, and the perfusion volume increased from 0.2ml to 0.7ml with age, as shown in Table 1. Then, without any experimental operation until 6 weeks of age, a mechanical stimulation of colorectal distention (CRD) was performed to enhance the establishment success rate and the stability of the model. The model preparation group were randomly divided into model group (model group), GV29 group, PC6 group, ST25 group, and ST36 group after modeling.

### 2.3. Electroacupuncture (EA)

Acupoint selection: GV29 (Yintang) is located at the middle point between two eyes [19], PC6 (Neiguan) about 3 mm to the wrist transverse stripe on the axopetal end, and ST36 (Zusanli) about 5 mm inferior of the capitulum fibulae and posterior-lateral to the hind-limb knee joint [20]; ST25 (Tianshu) [21] was approximately 5 mm away from the Shenque acupoint (the distance to subxiphoid area and pubic symphysis at a ratio of 8 : 5, 8 from the top and 5 from the bottom).

Acupuncture method: In addition to the blank group, rats in the model group were bound restraint 20min without acupuncture, and the treatment groups were routinely treated with EA at the age of 9 weeks. The rats in 4 EA groups were fixed by soft cloth sleeve, a needle was inserted into the acupoint area and connected with the positive pole, and another needle was inserted 1mm in the same area and connected with the negative pole of Han's acupoint nerve stimulator (HANS). The parameters of the HANS were as follows: sparse-dense wave with a frequency of 2/100 Hz and current of 0.1~0.3 mA. EA stimulation was performed 20 min per session, one treatment every other day, with a total of 5 sessions.

### 2.4. Abdominal Withdrawal Reflex (AWR)

The abdominal withdrawal reflex was used to assess the visceral sensitivity of the rats. Rats were fasted for 12h before assessment. Abdominal withdrawal method was improved by referring to Al-Chaer [22], who, using BL-420S biological function experimental system, collected intestinal pressure signals through pressure channel to observe the sensitivity of colon under different pressure. The BL-420S, pressure transducer, and microinjection pump were connected with three-way piece, and the pipeline was kept airtight. The microinjection pump (injected with 2.0ml purified water) was connected to a self-made saccule, which was coated with paraffin oil and was slowly inserted into the anus to a depth of about 8 cm. When the microsyringe pump was opening, the saccule was dilated by injecting water at a speed of 1ml/min for 90 seconds. The pressure signals were showed in BL-420S' display screen. When the rat's colon was contracted by stimulation, the pressure line would present a wave. The time before the first contraction wave appeared (latent period) and the number of contraction waves in 90s (contraction wave) was recorded. Each rat was detected 3 times, with each interval being at least 30 min, taking the average value.

### 2.5. Open-Field Test (OFT)

The next day after AWR test, open-field test reflecting the mental state of rats was performed. After adaption to the experimental environment for about 10min, the rats were put into the box of autonomous behavior device. The number of horizontal and vertical movement (two forelegs are erected at the same time for 1 time) times of the rats in the box within 5min was recorded by software. The open box was cleaned after testing every time, without any foreign matter or odor. Horizontal activity reflects the excitability of animals and vertical activity reflects the animals' curiosity of risk and uncertainty in the surrounding environment. In this way, animal emotion and psychology were evaluated.

### 2.6. Specimen Collection and Processing

After the OFT, all rats were sacrificed to collect specimens. ACC brain tissue was taken from subcortical and anterior fontanelle and ground in the EP tube with 0.5ml Trizol for real-time quantitative polymerase chain reaction (PCR). The colon was collected 1 cm above the anus from 7^th^ cm to 8^th^ cm and fixed by 10% neutral formalin.

### 2.7. Real-Time PCR

Total RNA was extracted with Trizol from ACC brain tissues. Promega ImProm-II Reverse Transcription System kit (Promega) was used to reverse transcription: RNA template 4ul, Oligo(dT)15 0.5ul, random primers 0.5ul, run cycle 70°C 5min and then static on ice10min; adding nuclease free water 4.5ul, 5xbuffer 4ul, MgCl_2_ 4ul, dNTP 1ul, RNasin ribonuclease inhibitor 0.5ul, ImProm-IITM reverse transcriptase 1ul, run cycle 25°C 5min, 42°C 1h, and 70°C 15min; _C_DNA template 3ul, SYBR Green PCR Master Mix 10ul, Primer1(20) 0.5ul, Primer2(20) 0.5ul, RNase free H2O 11ul, 94°C 5min, 94°C 30s, 59°C 30s, 72°C 60s, 35 cycles to amplification. Gene expression levels were measured by qRT-PCR using the following primer sequences: NMDA receptor 1:sense: 5-GCTGTACCTGCTGGACCGCT-3', antisense: 5-GCAGTGTAGGAAGCCACTATGATC-3', predicted size (bp): 219bp. NMDA receptor 2A: sense: 5'-TCCATTCTTCTGTCATCCTGC-3', antisense: 5'-AAGACCGTCTCTCACTCTTGC-3', predicted size (bp)225bp. NMDA receptor 2B: sense: TGCACAATTACTCCTCGACG-3', antisense: 5'-TCCGATTCTTCTTCTGAGCC-3', predicted size (bp): 222bp. The amplification curve and melting curve of real-time PCR were confirmed at the end of reaction. The Ct value of the sample was taken as the average value of three replicates. Relative quantification of target genes by ΔΔCt: the relative expression of the target gene = 2^−ΔΔCt^, ΔΔCt = experience group (target gene Ct- reference gene)- control group (target gene Ct- reference gene). The beta-actin was used as reference gene, group CON was used as control gene, and the relative quantity of each target gene was quantified.

### 2.8. Immunohistochemical Method

NR1 and NR2B in colon were detected using immunohistochemistry. The tissue sections were dewaxed and hydrated by xylene I, II and alcohol with different concentration and distilled water. Antigen retrieval was performed by heating samples in citric acid buffer in a microwave oven. After washing with PBS for 3 × 2min, specimens were treated with 3% H_2_O_2_ in the dark for 10 min to inactivate endogenous peroxidases and then washed with PBS solution 3 × 3min. Specimens were immersed in the diluted anti-NMDAR1 antibody or anti-NMDAR2B antibody and incubated at 4°C overnight. The specimens were placed at room temperature for 30min, washed with PBS solution 3 × 3min, and incubated with the secondary antibody at room temperature for 30min. After washing with PBS for 2 × 3min, DAB developing solution was added for observation under microscope. After the target indicated positive reaction, it was washed with distilled water to terminate the development. After being redyed with hematoxylin, differentiated with hydrochloric acid alcohol, and returned blue with ammonia liquor, they were dehydrated, mounted, and observed under a light microscope.

NMDAR immunoreactivity was determined in the mucosa and muscularis of the colon, and each tissue slice was photographed under a high power microscope with 2 visual fields randomly, with 12 eyes per group. The collected images were analyzed by using Image-Pro Plus 6 image analysis system; the positive expression of NMDAR immunoreactivity based on each area of view area (A) and integrated optical density (IOD) of the positive reaction were measured to calculate the average optical density (AOD, IOD/A) as the quantitative index level of NMDAR expression.

### 2.9. Statistical Analysis

Data were analyzed using the Statistical Package for the Social Sciences (SPSS) version 20.0, and all data were expressed as mean ± standard deviation ($\bar{X}\pm s$). If the data of each group are normal and the variance is homogeneous, they were analyzed by one-way analysis of variance (ANOVA), followed by LSD to compare between groups; if the groups were normal and the variance was not uniform, the Welch test was used; if each group did not obey the normal test, the nonparametric test was used, followed by the comparison between each 2 groups. A value of P<0.05 was considered to be statistically significant.

## 3. Experimental Results

### 3.1. Effects of EA on AWR

**Figure 1:**
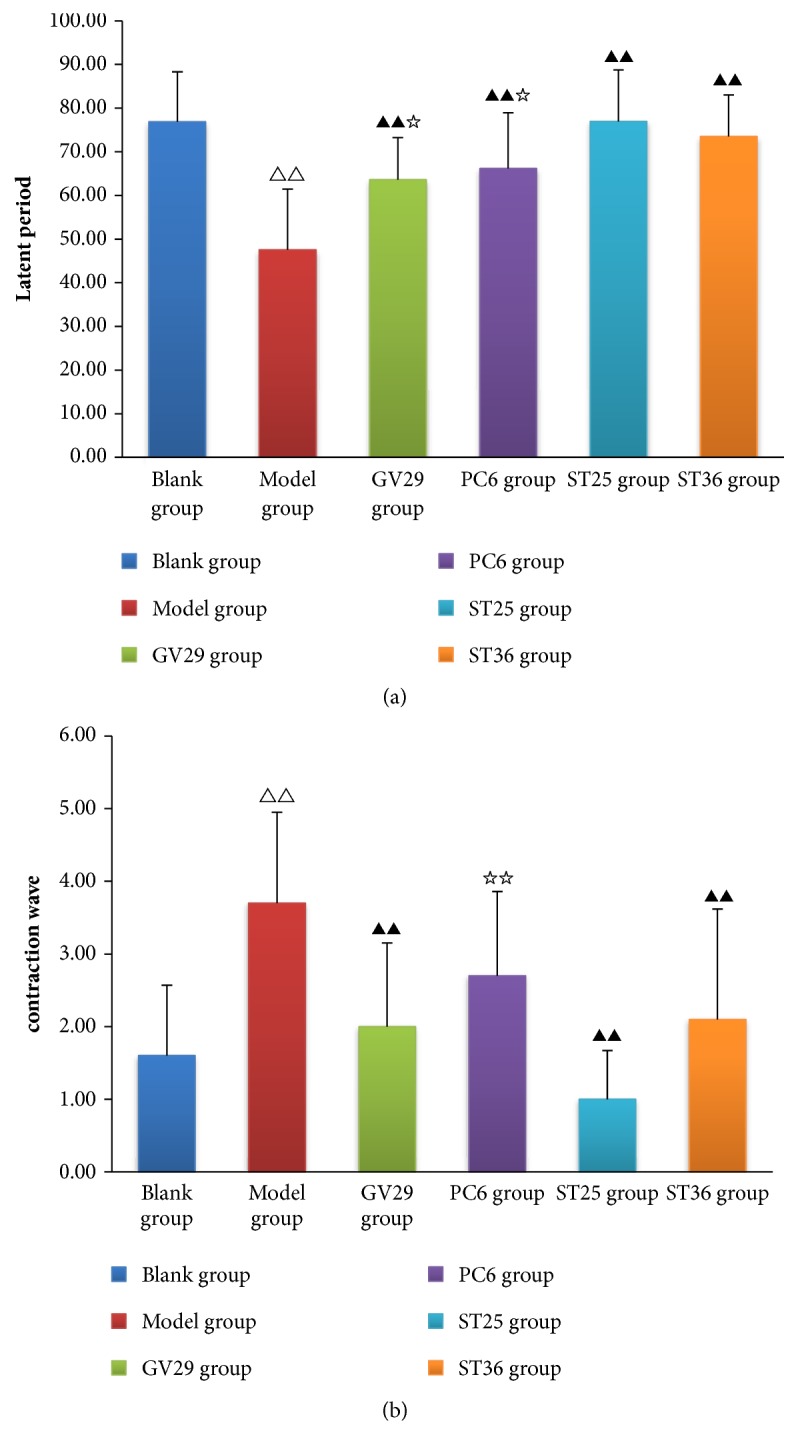
(a) Bar chart demonstrating the latent period, (b) bar chart demonstrating the contraction wave. Data are presented as mean ± SD (n=10). Note: ^△△^P<0.01, versus blank group; ^▲▲^P<0.01, versus model group; ^☆☆^P<0.01, ^☆^P<0.05, versus ST25 group.

In AWR experiment, the time before the first contraction wave appeared (latent period) and the number of contraction wave in 90s (contraction wave) reflected the intestinal sensitivity of rats. As shown in Figure 1, the latent period of IBS rats was significantly shortened and the number of the contraction waves increased significantly (P<0.01). After EA, the latent period was significantly prolonged (P<0.01) and contraction wave significantly decreased (P<0.01) in all EA groups except PC6 group. Comparison between groups: the latent period in ST25 group was longer than those in PC6 and GV29 group (P<0.05), and the contraction waves in ST25 group were fewer than those in PC6 group (P<0.01).

### 3.2. Effects of EA on OFT

**Figure 2:**
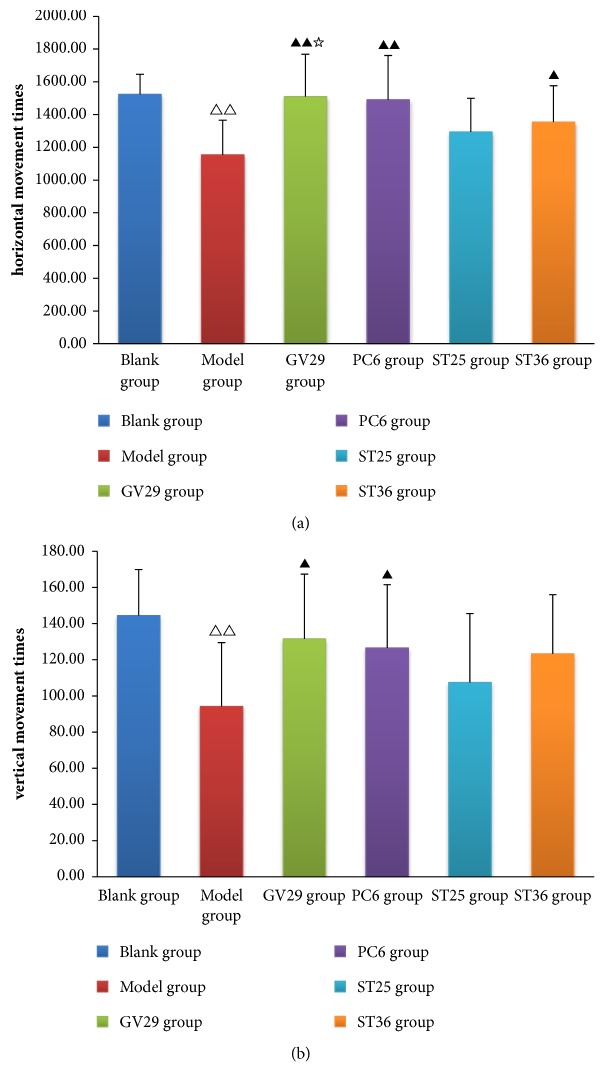
(a) Bar chart demonstrating the horizontal movement times, (b) bar chart demonstrating the vertical movement times. Data are presented as mean ± SD (n=10). Note: ^△△^P<0.01, versus blank group; ^▲▲^P<0.01, ^▲^P<0.05, versus model group; ^☆^P<0.05, versus ST25 group.

In OFT, the number of horizontal and vertical movement times could reflect depression or anxiety in rats. As shown in Figure 2, the number of horizontal and vertical movement times of IBS rats was significantly reduced (P<0.01) and increased after EA. Compared with the model group, in GV29 and PC6 group the number of horizontal movement times increased significantly (P<0.01) and vertical movement times increased (P<0.05); in ST36 group only horizontal movement times increased (P<0.05). Compared with the ST25 group, GV29 group has more number of horizontal movement times (P<0.05).

### 3.3. Expression of NR1, NR2A, and NR2B in ACC

**Figure 3:**
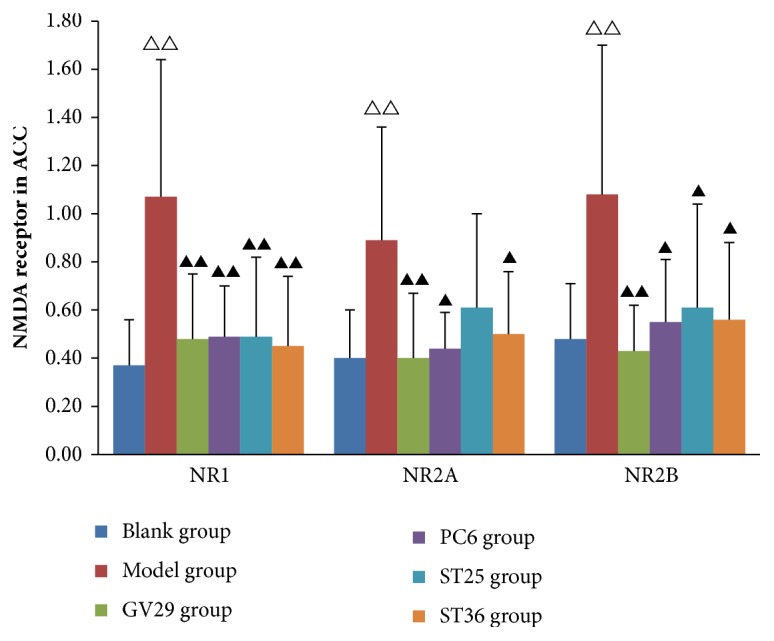
Bar chart demonstrating the NMDA receptor in ACC. Data are presented as mean ± SD (n=6). Note: ^△△^P<0.01, ^△^P<0.05, versus blank group; ^▲▲^P<0.01, ^▲^P<0.05, versus model group.

The above experiments showed that the NR1, NR2A, and NR2B in ACC played important roles in the pathogenesis of visceral pain and pain-emotion. In Figure 3, NR1, NR2A, and NR2B in ACC of IBS rats increased significantly (P<0.01) and decreased with different degree after EA at different acupoints. Compared with the model group, NR1 in ACC of all groups significantly reduced (P<0.01), and NR2A and NR2B significantly reduced in GV29 group (P<0.01) and decreased in PC6 group and ST36 group (P<0.05), while, only NR2B reduced in ST25 group (P<0.05).

### 3.4. Expression of NR1 and NR2B in Colon

**Figure 4:**
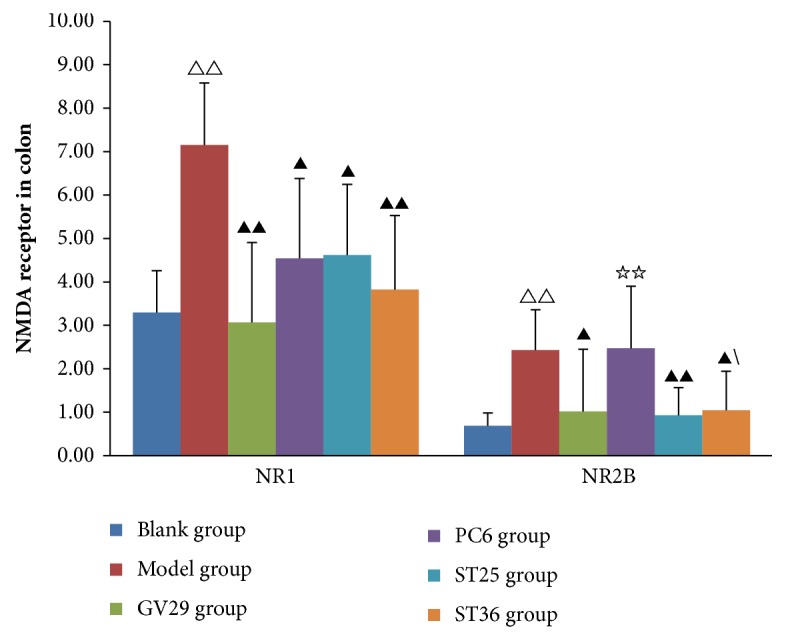
Bar chart demonstrating the NMDA receptor in colon. Data are presented as mean ± SD (n=6). Note: ^△△^P<0.01, versus blank group; ^▲▲^P<0.01, ^▲^P<0.05, versus model group; ^☆☆^P<0.01, versus ST25 group.

**Figure 5:**
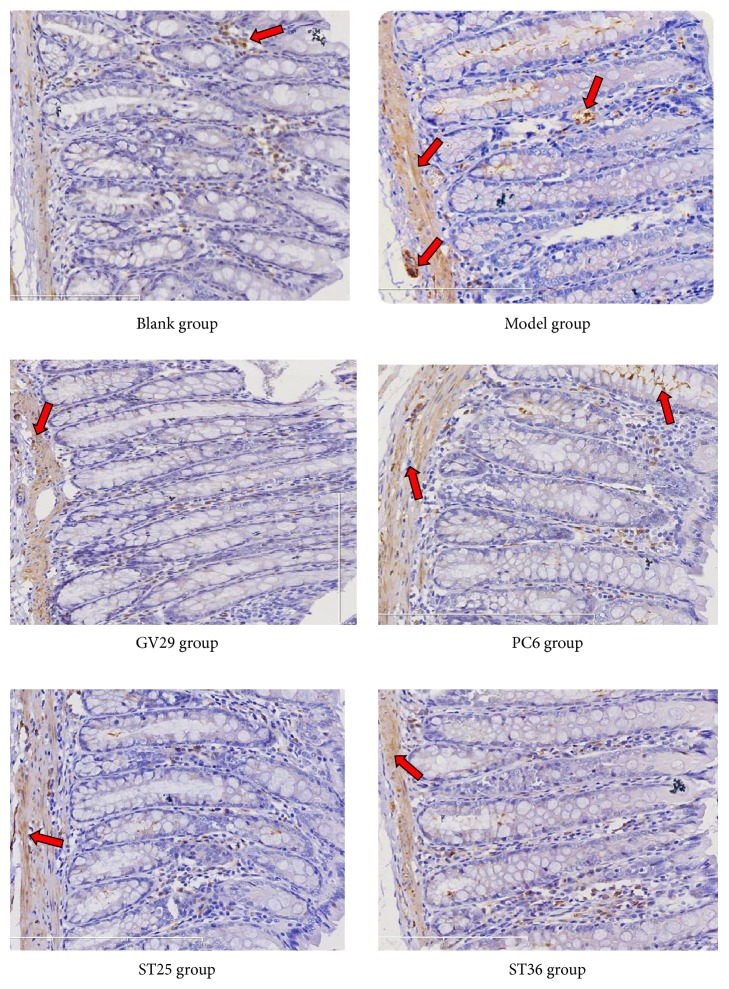
Representative photomicrographs showing immunohistochemical expression of NR2B (yellow or brown) in ACC (×200).

**Figure 6:**
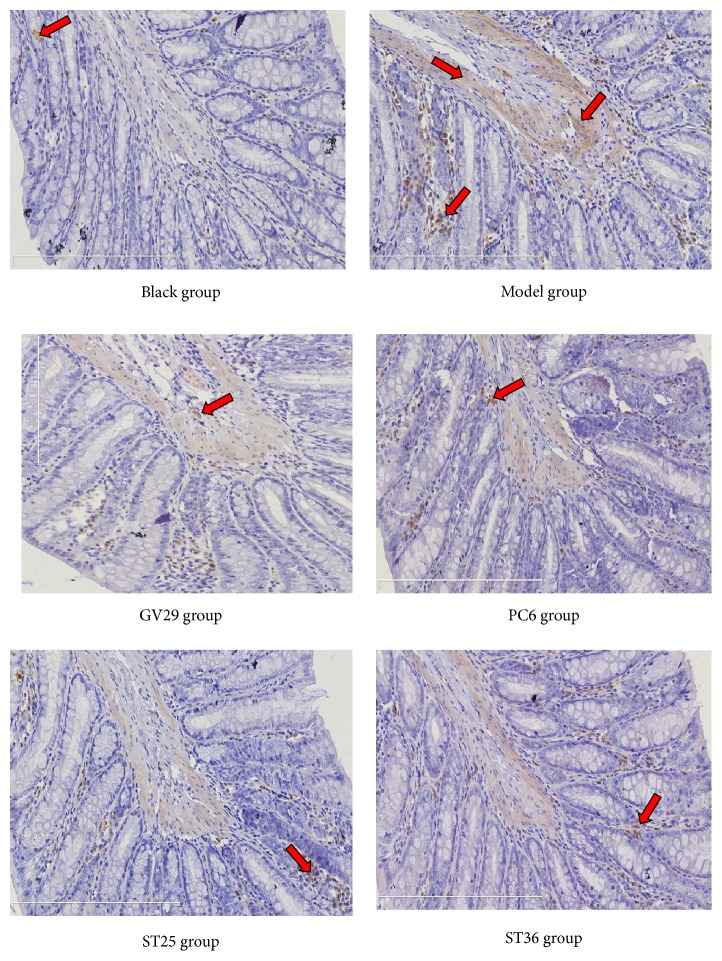
Representative photomicrographs showing immunohistochemical expression of NR2B (yellow or brown) in colon (×200).

NR1 and NR2B in colon also played important roles in the pathogenesis of visceral pain. In Figures 4, 5 and 6, NR1 and NR2B in the colon of IBS rats were significantly increased (P<0.01). After EA, NR1 in colon of GV29 and ST36 group decreased significantly (P<0.01) and decreased in PC6 group and ST25 group (P<0.05); NR2B in ST25 group significantly decreased (P<0.01) and decreased in GV29 and ST36 group (P<0.05). Meanwhile, NR2B in colon of PC6 group was significantly more than that in the ST25 group (P<0.01).

## 4. Discussion

Irritable bowel syndrome (IBS) is a functional gastrointestinal disease closely related to mental health. It has both physical symptoms and mental disorders. Abdominal withdrawal reflex [23], which can reflect the pain tolerance threshold and sensitivity of rats by different stimuli, indicates the visceral sensitivity of IBS model rats.

Open-field test [24] was a classic experiment based on the characteristic of rats being naturally close to the edge field (haptotaxis) in a new environment. Exploratory behavior and spontaneous activity in a new environment can be used to test the excitement or depression state in central nervous system of animals. In this experiment, the latent period of IBS rats was significantly shortened and contraction wave increased showing that the intestinal sensitivity was enhanced, and the horizontal and vertical movements in the open-field test were significantly reduced indicating that the rats were prone to depression. It shows that the experimental model is successfully prepared.

N-methyl-D-aspartic acid receptor [10, 11] (NMDA receptor) is a kind of monosodium glutamate receptor involving in essential brain functions like learning, memory formation and consolidation, mood, and behavioral responses to exogenous stimuli depending on the activity of NMDA receptors. NMDA receptor includes three subunits: NR1, NR2, and NR3; NR1 is the basic subunit of NMDA receptor [25], which is related to pain perception, NR2 (in the ACC is mainly NR2A and NR2B) is a regulatory subunit, and a single NR2 combination has no response to agonists. The activation of NMDA receptor in ACC plays an important role in the production of pain-emotion [26]. Study [27] has shown that NMDA receptors preferentially participate in the processing of emotional dimensions of pain in ACC and are closely related to negative emotions especially in rACC [26, 28]. Studies by Liu Zong et al. [29] suggest that ACC is an important component of the visceral pain response pathway, and the upregulation and activity enhancement of NMDA receptor in ACC can regulate the pain-emotion. Ren [30] found that noxious stimulation can induce the expression of NR2A and NR2B in the anterior cingulate cortex of rats and activate the Gly loci in NR1 to activate the ERK-CREB signaling pathway to form pain-emotion. NMDA receptor also played a regulatory role in ACC's pain sensitization. Huang [31] found that NR2A and NR2B in ACC increased after inflammation and play an important role in the formation of visceral sensitivity. Hence, NMDA receptor is not only involved in the formation of visceral sensitivity, but also closely related to pain-emotion. It is the key factor of visceral pain and pain-emotion.

Different from ACC brain region, Valle-Pinero et al. [32] found that NMDA receptor existed in the colonic mucosa and muscularis. By identifying NMDA receptor in the intestinal nervous system, it was found that NR1 coexisted with NR2B, but no NR2A was found. Qi Qingqing [33] also successfully detected NR1 and NR2B in the colon and confirmed that activation of NMDA receptor could induce the formation of IBS visceral sensitivity. In this experiment, the expression of NMDA receptor in the IBS model rats increased in both ACC and colon, which was consistent with the previous experimental results.

Acupuncture has a long history of analgesia and has a good effect on all kinds of pain, about 43 indications of acupuncture and moxibustion recommended by the WHO in 1987; almost half of the symptoms were associated with pain. Consensus Development Conference on Acupuncture, held in November 1997 by the National Institutes of Health of the United States, believed that acupuncture is an effective treatment with scientific basis. Acupuncture not only has good curative effect on visceral pain [34], but also has a positive effect on the emotion induced by pain [35, 36]. In this study, the abdominal sensitivity of IBS rats was decreased after EA at different acupoints, the behaviors tended to be normal and the mental and psychological abnormality were improved. It is consistent with the previous research results [37, 38].

The results of this study showed that all acupoints were effective for IBS; however, each point had different effect on different IBS related symptoms. According to the classical theory of acupoints, GV29 (Yintang) is good at tranquilizing, ST25 (Tianshu) is mainly used for regulating bowel and relieving abdominal pain, and PC6 (Neiguan) and ST36 (Zusanli) are good for both physical and mental disease. GV29 belongs to Governor Vessel, which goes into the brain and its functions are closely related to the brain. The clinical studies [39, 40] found that “GV29” could regulate Governor Vessel and relieve the symptoms of insomnia, anxiety, and depression in patients with liver disease; meanwhile, GV29 also had certain curative effect on IBS [41]. Another study [42] has demonstrated that the psychiatric disorder-related cerebral functional regions, such as the frontal lobe, cingulate gyrus, and cerebellum, could be activated by stimulating GV29. The mechanism may be that acupuncture GV29 point after nerve impulses can be directly into the brain resulting in sedative effect caused by 5-TH, NE, BDNF, and other neurotransmitter release [43], thus creating a tranquilizing effect. PC6 has the effect of calming the mind due to the close relationship between the pericardium and the heart. Moreover, PC6 is the* Luo*-connecting point of pericardium meridian to connect triple energizer meridian, which could rectify Qi to regulate the function of intestine. Acupuncture on PC6 has a therapeutic effect on depression and anxiety [44] and can relieve the damage of astrocytes caused by chronic stress and play an antidepressant role possibly by upregulating the expression of GFAP in prefrontal cortex [45]. Li Wenwen et al. [46] found that the corresponding changes of brain waves occurred in the frontal lobe and cingulate cortex area of brain when stimulating PC6 point, and the central alpha wave energy was enhanced after stimulating PC6 point. Modern experimental research [47] had proved that acupuncture at PC6 can accelerate gastric emptying and improve dyspepsia and has an auxiliary therapeutic effect on IBS [48]. Therefore, applying EA at PC6 point not only can calm mind, but also can adjust the intestine, regulating the body and the psychic simultaneously. ST25, “the Front-Mu point” of large intestine is the main source of Qi and blood in the large intestine, and it is the first choice for treating intestinal diseases. Liu et al. [49] found that acupuncture at ST25 has bidirectional regulation effect on intestinal movement and has good curative effect on constipation and diarrhea. ST25 is one of the most commonly used acupoints to treat diarrhea-predominant IBS [50]. ST36 point is the “lower He-Sea point” of stomach and good at treating gastrointestinal diseases [51, 52]. EA at ST36 restores the rectal distension induced impairment in both colonic contraction and transit by enhancing vagal activity mediated via the cholinergic pathway [53]; EA ST36 also attenuates hemorrhage-induced intestinal inflammatory insult and protects the intestinal barrier integrity [54]. In addition, study found that EA at ST36 could relieve depression [55] and the effect of relieving the depressive behavior of rats was related to its pertaining meridian [56]. Wu et. al [56] found that electroacupuncture at ST36 can regulate the expression of *β*CaMKII in the lateral habenular nucleus and the protein of hippocampal brain derived neurotrophic factor (BDNF) to relieve depression.

Modern research [57] shows that the indication function of acupoints is related to the spinal segment of the acupoint and acupuncture has a reflex neuromodulation effect on viscera. Acupoints controlled by different spinal segments can regulate viscera, but their effects are different [58]. The upper nerve of the trochlear dominated the superficial skin and the temporal branch of the facial nerve dominated the muscle layer of GV29 in human body. Acupuncture at GV29 point can directly introduce impulses into hypothalamus, hippocampus, and prefrontal cortex, which are closely related to emotion [59]. Wang Jinjin et al. [60] observed the muscles distribution of acupoints in distal limbs of twelve meridians and found that PC6 is controlled by C7 ~ T1 nerves, ST36 mainly controlled by L4~S1 nerves, and ST25 controlled by T9~T11 nerves. The nerves derived from T10-L3 and the sacral plexus govern the large intestine. Thus, the spinal segment of the large intestine overlaps with that of ST25 and is adjacent to that of ST36. The study [61] demonstrates that the acupoint specificity existing in acupuncture relieves visceral hypersensitivity and the effects are more predominant at the acupoints on stomach meridian innervated by the same or adjacent spinal segments. Chen Xiaoman [62] found that the analgesic effect of local acupoint extraction on IBS abdominal pain is superior to distal acupoint, and the possible mechanism might be that there are more overlaps of spinal segments controlling viscera and nearby local points. After acupuncture, the regulating effect on gastrointestinal motility is achieved through the body surface-sympathetic reflex pathway [63]. Therefore, the analgesic effect of acupuncture at ST25 is more obvious. So according to the spinal segment domination of the acupoint, GV29, a point on the head, has better effect in regulating mood and ST25 located in the abdomen is better in relieving abdominal pain, while PC6 and ST36 have their own specific effects and indications. This is consistent with the results of this study.

The results show that the acupoint effect is specific, but this specificity is relative. Chinese medicine regards the human body as a whole. It believes that there is interaction between viscera and emotion, and the same among viscera. Which is consistent with “biopsychosocial” medicine mode [64], “psychosomatic disease”, and “brain-gut axis” [65]. Due to the organism being considered as a whole, EA at acupoints located in different parts can alleviate IBS abdominal pain and relieve abnormal emotion by changing the internal environment. However, the specificity of acupuncture effect is relative. The reason is that the regulatory intensity of the same neurotransmitter receptors is different in different tissues. The results of our study provide some experimental evidence for the specificity of acupuncture points, which is one of the important guiding principles for point selection in clinical treatment.

## Figures and Tables

**Table 1:** 0.5% acetic acid enema dose of colorectal in infancy rats.

Age (d)	8	9	10	11	12	13	14	15	16	17	18	19	20	21

Dose (ml)	0.2	0.2	0.2	0.3	0.3	0.3	0.4	0.4	0.5	0.5	0.6	0.6	0.7	0.7

## Data Availability

The data used to support the findings of this study are available from the corresponding author upon request.
